# Type 2 diabetes mellitus in Bangladesh: a prevalence based cost-of-illness study

**DOI:** 10.1186/s12913-019-4440-3

**Published:** 2019-08-27

**Authors:** Afsana Afroz, Khurshid Alam, Liaquat Ali, Afsana Karim, Mohammed J. Alramadan, Samira Humaira Habib, Dianna J. Magliano, Baki Billah

**Affiliations:** 1 Department of Epidemiology and Preventive Medicine, School of Public Health and Preventive Medicine, Monash University, 553 St. Kilda Rd., Level 4, Melbourne, VIC 3004 Australia; 2 School of Population and Global Health, The University of Western Australia, Perth, Australia; 3 Bangladesh University of Health Sciences (BUHS), Dhaka, Bangladesh; 4 Bangladesh Institute of Research and Rehabilitation in Diabetes Endocrine and Metabolic Disorders (BIRDEM), Dhaka, Bangladesh; 5 BakerIDI Heart and Diabetes Institute, Melbourne, Australia

**Keywords:** Burden of diabetes, Cost-of-illness, Direct cost, Indirect cost, Management plan, Type 2 diabetes

## Abstract

**Background:**

The economic burden of type 2 diabetes has not been adequately investigated in many low- and lower middle-income countries, including Bangladesh. The aim of this study was to estimate the cost-of-illness of type 2 diabetes and to find its determinants in Bangladesh.

**Methods:**

A cross-sectional study was conducted in 2017 to recruit 1253 participants with type 2 diabetes from six diabetes hospitals, providing primary to tertiary health care services, located in the northern and central regions of Bangladesh. A structured questionnaire was used for face-to-face interviewing to collect non-clinical data. Patients’ medical records were reviewed for clinical data and hospital records were reviewed for hospitalisation data. Cost was calculated from the patient’s perspective using a bottom-up methodology. The direct costs for each patient and indirect costs for each patient and their attendants were calculated. The micro-costing approach was used to calculate direct cost and the human capital approach was used to calculate indirect cost. Median regression analysis was performed to identify the determinants of average annual cost.

**Results:**

Among the participants, 54% were male. The mean (±SD) age was 55.1 ± 12.5 years and duration of diabetes was 10.7 ± 7.7 years. The average annual cost was US$864.7 per patient. Medicine cost accounted for 60.7% of the direct cost followed by a hospitalisation cost of 27.7%. The average annual cost for patients with hospitalisation was 4.2 times higher compared to those without hospitalisation. Being females, use of insulin, longer duration of diabetes, and presence of diabetes complications were significantly related to the average annual cost per patient.

**Conclusions:**

The cost of diabetes care is considerably high in Bangladesh, and it is primarily driven by the medicine and hospitalisation costs. Optimisation of diabetes management by positive lifestyle changes is urgently required for prevention of comorbidities and complications, which in turn will reduce the cost.

**Electronic supplementary material:**

The online version of this article (10.1186/s12913-019-4440-3) contains supplementary material, which is available to authorized users.

## Background

Diabetes is one of the most prevalent non-communicable diseases globally and, currently, the disease is a major public health issue in developing countries because of its chronic nature, rapidly increasing prevalence, related complications, and the requirement of long-term care. The higher prevalence of diabetes is related to an increased prevalence of obesity, population ageing, population growth, urbanisation and physical inactivity [1]. The International Diabetes Federation (IDF) estimated that, worldwide, approximately 425 million people had diabetes in 2017; the number is projected to be 629 million by 2045. For treating and preventing diabetes and its related complications, an estimated US$727 billion was spent in 2017, which represented an 8% increase from that estimated for 2015. The cost has been projected to be US$776 billion by 2045 [2]. The annual cost for people with diabetes is mainly related to direct (e.g. cost for medicine, hospital care, laboratory tests, etc.) and indirect costs (e.g. productivity loss from disability, premature mortality, etc.) [3].

Compared to people living in high-income countries, people in low- and lower middle-income countries (LMICs) have a lack of access to health insurance or publicly available medical services. Thus, they pay a larger share of out-of-pocket (OOP) health expenditures. Furthermore, in some LMICs, people with diabetes and their families bear almost all of the expenditure related to diabetes care [4]. The prevalence of diabetes has escalated more rapidly in South East Asia than in any other large region in the world [2]. Literature showed that about 90 to 95% of all diagnosed diabetes cases of this region are type 2 diabetes [5, 6]. In Bangladesh, the estimated prevalence of diabetes among adults was 9.7% in 2011 [7] and the number is projected to be 13.7 million by 2045 [2]. According to the Bangladesh National Health Accounts, in 2010, Bangladesh spent US$2.3 billion on health (or US$16.20 per person per year) and 64% of this cost came from OOP payments [8]. However, according to the World Health Organization (WHO), in 2014, Bangladesh spent US$88 per person per year on health [9]. It has been observed that, on average, a household spent 7.5% of its total income on receiving health care, with the poorest 20% of the households spending approximately 13.5% of their income on it [8]. The per capita gross domestic product (GDP) of Bangladesh was US$1677 in 2018 [10], and nearly one-third (31.5%) of the population in the country was below the poverty line [11]. Hence, the OOP health care expenditure posed a notable economic burden on the Bangladeshi population.

In high-income nations, such as the USA [12–15] and in some European [16] and upper middle-income countries [17, 18], the economic burden of diabetes is well-acknowledged and investigated. Most of these studies have estimated the economic burden in terms of cost, while others [14, 15, 17] investigated the factors (e.g. patients’ demographics, complications, payment methods and health care utilisation) correlated with the total cost. Low- and lower middle-income countries represent 80% of the global diabetic population [2]; however, research-based evidence on diabetes management-related cost is limited for most of these countries, including Bangladesh. A study [19] in Bangladesh that addressed the cost and its determinants recruited a relatively small sample from a single hospital located in the capital city, and thus covered mostly urban residents. Furthermore, the cost was calculated from the outpatient department only, which may underestimate the average annual cost.

Thus, the aim of this study was to estimate the average annual cost and to find its determinants, where the cost data included both outpatients and hospitalisation. This study’s findings will provide the most up-to-date information on the economic burden incurred by people with type 2 diabetes mellitus (T2DM) in Bangladesh, which will be useful as an important aid in the planning of health care needs and allocation of scarce resources.

## Methods

### Study design and study population

**Fig. 1:**
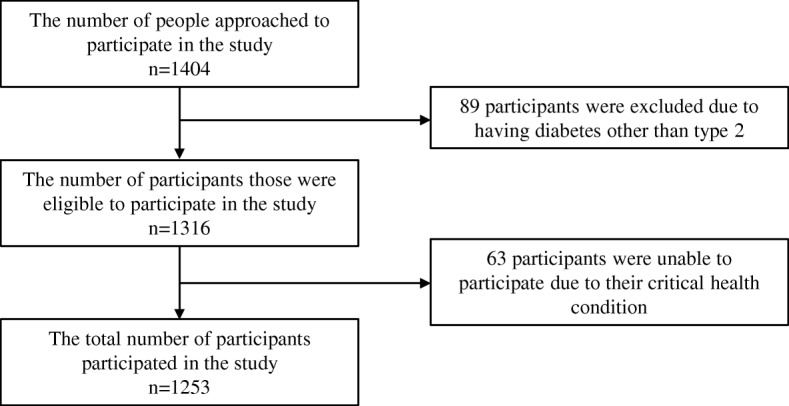
Study participant’s recruitment flowchart

A cross-sectional study was conducted with a prevalence-based approach [20]. Data was collected from six hospitals (specialising in diabetes) where patients’ records were available for the previous years, located in the northern and central regions of the country. Two of these hospitals are from the central region, providing primary to tertiary health care, particularly to urban residents. The remaining hospitals are from the northern region, two of them providing primary and secondary health care and the other two providing primary to tertiary health care to people residing in semi-urban and rural locations. Patients attending hospitals providing primary and secondary but in need of tertiary care are usually referred to the tertiary care hospitals. Due to a similar social and economic status for people living in the northern and southern regions, the patients from the hospitals of the northern region are comparable to those living in the southern region of the country. Thus, no hospitals were selected from the southern region. The Diabetic Association of Bangladesh (BADAS), a not-for-profit but mostly self-sustaining social welfare organisation, directly or indirectly (through affiliated local associations) owns all the selected hospitals. BADAS, the highest diabetes care provider, has 75 diabetic centres/hospitals which cover all 64 districts (second highest level tire of regional administration) across the country. Due to lack of adequate services related to diabetes in public hospitals, particularly in peripheral areas, majority of the people with diabetes are treated and managed by the hospitals under BADAS. The hospitals were purposively selected to ensure that the study included patients from rural-urban as well as professionally mixed populations (e.g. service holders, businessmen, farmers, day labours, housewives, etc.) attending various levels of health care services. Between April and September 2017, 1253 participants were recruited using systematic random sampling and probability proportional to size (PPS) methods (Fig. 1). The target population comprised registered adults of either gender with a minimum one-year duration of T2DM. People with other types of diabetes or who were pregnant at the time of data collection were excluded as those people may have some additional expenses other than T2DM. A team of trained data collectors was involved in the data collection. At the beginning of the interview, the data collectors provided an explanatory statement to each participant and, upon his/her agreement to participate by signing the consent form, participants were interviewed face-to-face. Participants were recruited from the outpatient department of the selected hospitals and those who were referred to hospital admission were followed-up to collect their hospitalisation information from the hospital inpatient department.

### Data collection instrument

A structured questionnaire (Additional file 1) was developed and used in a secured web-based application, Research Electronic Data Capture (REDCap), for data collection [21]. Prior to the main survey, the content validity of the questionnaire was evaluated and pretested, using a pilot study conducted in a tertiary care hospital. The questionnaire gathered each patient’s details and demographics, diabetes-related information, cost-related information and the patient’s lifestyle behaviour. Patients’ medical records (guide books) were also reviewed to retrieve information pertaining to clinical status and the number of medical services received during the previous year. To obtain information about the types and quantities of currently prescribed medicine, a copy of the prescription was kept in the REDcap application as an image for further use.

### Calculation of costs

The total cost of T2DM was calculated from the patient’s perspective for the year 2017, considering direct and indirect costs as the major components. Direct cost was estimated using a bottom-up approach for primary data collection [22] and divided into the two following sub-categories: direct medical costs, which comprise the costs of hospitalisation, outpatient visits, medicine, laboratory tests, and other service utilisation (including the use of self-monitoring blood glucose and consumables); and direct non-medical costs, which comprise the cost of transportation and meals en- route to the hospital.

The micro-costing approach was used to identify cost items in as much detail as possible for calculating direct cost [23]. Cost per daily dose [24] of each medicine and therapy was defined. Costs related to medicine, consultancy and laboratory tests that patients paid OPP were collected from a tertiary level hospital located in the central region. It should be noted that the health insurances support is literally non-exists in Bangladesh, thus all payments met by OPP. All hospitals under the BADAS are homogeneous; thus, there is a negligible cost variation between the selected hospitals. For other components of direct medical cost and direct non-medical cost, each participant’s responses to the questionnaire were considered as a reference. Each component of direct cost was calculated by multiplying the unit cost with the quantities of medical services received during the previous year. The cost of hospitalisation (including hospital stay, medicine and laboratory tests during the stay) was retrieved for each patient from their hospital record, which was provided by the accounts department of the hospital. The total direct cost was calculated by adding up all components of direct medical and direct non-medical costs.

The indirect cost was calculated for both patients and their attendants’ en-route to the hospital. The productive time lost to attend outpatient visits and during hospital admission was recorded based on the information provided by the patients and their attendants. The human capital approach [25] was used to calculate the indirect cost for those who were productive and in the formal workforce or housewives, but not for people who were unable to work (retired or ill health) or who chose not to work. The productivity loss of housewives was calculated using the minimum wage rate of Bangladesh (US$224/annum) [26] as well as the median income of the participants who were in the formal workforce. The total cost was calculated by adding up total direct and total indirect costs.

All costs were calculated in Bangladeshi currency, Taka (BDT) and then, to add an international perspective, they were converted into US$ using the mid-year currency conversion rate for the year 2017 (US$1 = BDT80).

### Ethical approval

The study has been approved by the Monash University Human Research Ethics Committee (ID: 1469), the Ethical Review Committee of the Bangladesh University of Health Sciences (BUHS) and the Ethical Review Committee of the BADAS.

### Statistical analysis

Descriptive statistics includes mean with standard deviation for numerical data and frequency with percentage for categorical data. A normality test of cost data was performed using histogram, Q-Q plot and the Shapiro-Wilk test. Cost data was right skewed; hence, mean and median with percentiles was used for reporting it. A median regression was run to determine the factors related to average annual cost. A bootstrapping method was used to calculate the 95% confidence interval (CI) of regression coefficients [27]. A one-way sensitivity analysis was performed to evaluate the assumption that the use of minimum wage to calculate the indirect cost of housewives may give the lowest estimate. A two-way sensitivity analyses were undertaken to explore the change in average annual cost with assumptions of a 25% (+/−) change in the prevalence of insulin use and 25% (+/−) change in the prevalence of T2DM related complications. The statistical software package STATA SE version 15.0 was used for data analysis and a *p*-value of 0.05 or less was considered to be statistically significant.

## Results

### General characteristics of the study participants

The general characteristics of the study participants are presented in Table 1. Among the 1253 participants with T2DM, 681 (54.3%) were male. The mean age of patients was 55.1 ± 12.5 years. Approximately 45% of participants had a secondary level education and 23% had a tertiary level education. About two-fifths (40.5%) of the participants were employed and about a quarter (23.8%) were housewives. Three-quarters (73.2%) of the participants resided in urban areas and 51.2% of participants had a median monthly household income of US$375 (BDT30,000). Mean duration of diabetes was 10.9 ± 7.7 years and 43.5% of the participants had diabetes for more than 10 years. More than half of the participants (58.6%) managed diabetes by a combination of an oral hypoglycaemic agent (OHA) and insulin, 34.5% by OHA only (merging 1.8% of people with lifestyle modification with OHA only), and 6.9% by insulin only. More than half (55.9%) of the participants moderately adhered to medication, followed by 37.2% with high adherence, with only 6.9% having poor adherence. About one-third (34.6%) of the participants had family history of diabetes; only 19.8% had fair (HbA1c 7–7.9%) and 62% had poor (HbA1c ≥ 8%) glycaemic control. About half (48.9%) of the participants had up to two diabetes-related complications (coronary artery disease, stroke, diabetic foot, nephropathy, retinopathy and neuropathy) and 14.5% had three or more complications. The study results showed that 41.8% had hypertension, 12% had dyslipidaemia, and 22.6% had both. The mean productive time lost during outpatient visits was 7.3 ± 1.5 h per month. For patients with a history of hospitalisation, productive time lost was 10.4 ± 8.8 days per year (data is not shown in the table).

**Table 1:** General characteristics of the study participants

Variables	*n* (%) (*n* = 1253)
Gender	
Male	681(54.4)
Female	572 (45.6)
Age (mean ± SD)	55.1 ± 12.5
≤ 40	176 (14.05)
41–60	669 (53.39)
61–80	380 (30.33)
≥ 80	28 (2.23)
Education	
Illiterate	161 (12.8)
Primary	239 (19.1)
Secondary	566 (45.2)
Tertiary	287 (22.9)
Work status	
Unemployed	36 (32.8)
Employed	411 (40.5)
Housewives	508 (23.8)
Retired	298 (2.9)
Area of residence	
Rural	174 (13.9)
Semi-urban	162 (12.9)
Urban	917 (73.2)
Monthly household income (US$)	
≤ 250	447 (35.7)
251–750	497 (39.7)
751 and above	309 (24.6)
Duration of diabetes (in year)	
≤ 5	360 (28.8)
6–10	348 (27.7)
≥ 11	545 (43.5)
Mode of treatment	
OHA	432 (34.5)
Insulin	87 (6.9)
Insulin + OHA	734 (58.6)
Family history of diabetes	734 (58.7)
Yes	433 (34.6)
No	820 (65.4)
HbA1c (%)	
Good (≤6.9)	182 (18.2)
Fair (7–7.9)	198 (19.8)
Poor (≥8)	621 (62.0)
Number of complication^a^	
None	458 (36.6)
One or two	613 (48.9)
Three or more	182 (14.5)
History of co-morbidity	
None	296 (23.6)
Hypertension	524 (41.8)
Dyslipidaemia	151 (12.0)
Hypertension + dyslipidaemia	283 (22.6)

*OHA* Oral hypoglycaemic agent

^a^Complications include coronary artery disease, stroke, diabetic foot, nephropathy, retinopathy and neuropathy

### Cost-of-illness by socio demographic and clinical characteristics

Cost-of-illness (total cost) by socio-demographic and clinical characteristics is presented in Table 2. The results showed that for each variable, direct cost is higher compared to indirect cost. The average annual cost increased with the increasing age, which ranged from US$588 for aged <=40 years to US$1434 for aged > = 80 years (*p* < 0.001). Illiterate people spent the lowest (US$637) and that was highest (US$962) for people with up to secondary level education (*p* = 0.004). The average annual cost was higher for retired people (US$1062, *p* = 0.001) compared to unemployed people (US$676). People residing in rural areas (US$422) spent less compared to people living in urban areas (US$1024, *p* < 0.001), and the high-income group spent more (US$1062, *p* < 0.001) than the low-income group. The average annual cost increased progressively with the increased duration of T2DM (*p* < 0.001) and people with diabetes duration of more than 10 years spent US$1160.8 per year. The average annual cost for insulin users with a combination of OHA was US$1042.8 compared to US$526.2 for only OHA users (*p* < 0.001). As the number of complications increased, the average annual cost increased (*p* < 0.001). People with the presence of three or more complications spent US$1351.5 annually compared to US$532.2 for people without any complication. Likewise, people with the presence of both hypertension and dyslipidaemia had an average annual cost of US$1022.6 compared to that of US$659.4 for those with no comorbidity (*p* < 0.001).

**Table 2:** Details of annual cost in US$ by socio-demographic and clinical characteristics of the study participants

Variables	Direct cost		Indirect cost		Total cost	
	Mean	Median (percentiles)	Mean	Median (percentiles)	Mean	Median (percentiles)
Gender^b^						
Male	795.2	456.1 (273.6, 893.4)	81.6	19.5 (0, 62.5)	876.9	497.9 (292.0, 977.3)
Female	765.7	446.6 (278.8, 905.2)	84.4	52.1 (16.1, 93.7)	850.1	516.89 (332.7, 988.3)
Age (years)^c^						
≤ 40	519.6	331.1 (205.4, 564.9)	68.8	41.6 (18.48, 72.91)	588.4	385.7 (241.0, 616.2)
41–60	738.7	421.1 (270.3, 784.7)	103.0	52.1 (19.5, 104.2)	841.8	476.2 (310.1, 860.1)
61–80	934.9	587.4 (337.1, 1168.9)	56.1	0 (0, 52.1)	991.0	613.7 (351.3, 1247.3)
> 80	1376.9	1052.1 (682.9, 1845.5)	57.1	0 (0, 0)	1434.1	1133.1 (712.2, 1936.1)
Education^c^						
Illiterate	575.3	421.1 (254.1, 705.5)	61.6	39.1 (3.6, 72.9)	636.9	437.2 (287.5, 775.3)
Primary	759.5	513.1 (291.3, 958.0)	59.3	31.2 (0, 72.9)	818.9	536.41 (323.5, 1036.1)
Secondary	874.1	483.1 (294.7, 989.4)	88.0	35.1 (0, 83.3)	962.1	529.2 (333.7, 1092.1)
Tertiary	733.9	409.4 (224.6, 831.8)	104.5	41.6 (0, 91.1)	838.4	452.0 (261.2, 913.5)
Work status^c^						
Unemployed	627.5	469.0 (298.7, 598.3)	48.0	0 (0, 0)	675.6	469.0 (298.7, 606.2)
Employed	598.9	343.1 (217.8, 607)	116.0	41.6 (18.7, 87.5)	715.0	393.3 (255.3, 700.7)
Housewives	798.9	488.3 (294.3, 936.7)	87.0	52.1 (20.8, 104.1)	885.9	533.6 (359.3, 1025.1)
Retired	1027.0	617.4 (369, 1189.9)	34.8	0 (0, 10.9)	1061.9	644.3 (374.2, 1298.3)
Area of residence^c^						
Rural	359.8	291.3 (202.4, 442.2)	61.7	47.9 (11.4, 83.3)	421.6	369.0 (239.6, 522.3)
Semi-urban	388.3	295.5 (228.1, 494.6)	48.1	33.2 (7.8, 70.3)	436.5	353.3 (253.7, 541.1)
Urban	931.3	545.3 (324.9, 1089.6)	93.1	34.3 (0, 87.5)	1024.4	590.3 (361.4, 1205.4)
Monthly household income (US$)^c^						
≤ 250	739.6	465.5 (258.1, 846.1)	65.2	31.2 (0, 78.1)	804.9	499.4 (289.5, 920.3)
251–750	692.1	407.9 (267.6, 757.3)	64.6	41.66 (1.30, 72.9)	756.8	456.5 (305.2, 810.7)
751 and above	986.8	571.8 (340.5, 1262.8)	138.0	41.6 (0, 125.0)	1124.9	499.4 (289.5, 920.3)
Duration of diabetes (in year)^c^						
≤ 5	526.3	325.8 (220.8, 539.2)	53.7	32.6 (10.4, 67.1)	580.1	378.9 (260.2, 589.0)
6–10	645.9	398.0 (258.3, 691.7)	61.4	41.6 (3.9, 72.9)	707.4	458.1 (292.5, 760.6)
≥ 11	1044.1	679.8 (378.8, 1251.3)	116.7	37.5 (0, 114.5)	1160.8	746.6 (418.3, 1442.5)
Mode of treatment^c^						
OHA	476.2	291.4 (209.6, 511.2)	49.9	31.2 (4.0, 62.5)	526.17	335.0 (238.7, 534.3)
Insulin	702.6	441.9 (224.3, 842.3)	67.2	41.6 (8.8, 83.3)	769.89	477.1 (259.8, 902.8)
Insulin + OHA	970.9	586.0 (368.9, 1129.6)	104.2	41.6 (0, 104.1)	1075.20	642.7 (412.4, 1223.7)
Family history of diabetes^b^						
Yes	818.4	487.3 (287.0, 964.3)	97.1	41.6 (9.8, 93.7)	915.6	524.3 (321.7, 1100.8)
No	762.4	441.4 (270.5, 879.9)	75.4	31.2 (0, 72.9)	837.9	493.1 (298.9, 954.2)
HbA1c (%)						
Good (≤6.9)	527.1	318.9 (205.9, 513.3)	84.5	31.2 (0, 65.1)	611.7	367.5 (234.2, 562.8)
Fair (7–7.9)	552.1	366.6 (242.3, 637.5)	53.2	26.0 (0, 62.5)	605.4	409.9 (277.8, 665.1)
Poor (≥8)	567.9	398.9 (267.2, 633.2)	58.5	36.4 (7.8, 72.9)	626.5	450.4 (300.7, 717.3)
Number of complication^a,c^						
None	466.8	295.9 (207.3, 507.0)	56.3	31.2 (15.6, 65.1)	523.2	347.9 (237.8, 549.2)
One or two	657.5	379.9 (261.8, 670.7)	81.9	36.4 (0, 83.3)	739.4	437.1 (293.5, 739.7)
Three or more	1234.3	966.9 (511.1, 1519.8)	117.1	43.7 (0,130.2)	1351.5	1036.8 (547.8, 1718.5)
History of co-morbidity^c^						
None	595.5	354.2 (219.8, 580.7)	63.8	31.2 (10.4, 72.9)	659.3	418.2 (260.7, 632.8)
Hypertension	870.1	533.9 (317.5, 1041.9)	94.3	41.6 (0, 91.1)	964.4	582.4 (362.7, 1150.3)
Dyslipidaemia	566.8	332.6 (228.1, 561.9)	56.9	27.3 (9.1, 62.5)	623.7	376.1 (259.6, 608.8)
Hypertension + dyslipidaemia	926.9	534.0 (313.7, 1041.3)	95.7	41.6 (0, 91.1)	1022.6	587.6 (365, 1143.5)

*OHA* Oral hypoglycaemic agent, *HTN* Hypertension

^a^Complications include coronary artery disease, stroke, diabetic foot, nephropathy, retinopathy and neuropathy. ^**b**^Mann Whitney U test and ^c^Kruskal Wallis test were done for group comparison; *p*-value was considered significant at *p* < 0.05

### Annual cost-of-illness (COI) of diabetes care

Table 3 presents the estimated average annual cost per person by components of direct and indirect costs. The average annual cost of diabetes care was US$864.7, of which the direct cost was 90.5% with a mean of US$781.7 and the indirect cost was 9.5% with a mean of US$82.9. Without hospitalisation, the average annual cost was US$409.8, which increased to US$1705.2 with hospitalisation. Furthermore, Table 3 shows that of the overall direct cost, direct medical and non-medical costs were 96.9 and 3.1%, respectively. The medicine cost accounted for the largest share (60.7%) of overall direct cost followed by the hospitalisation cost (27.7%). Medicine cost was also the highest source of direct cost (83.5%) for patients without hospitalisation. For patients with hospitalisation, medicine cost contributed 50.7% of direct cost followed by a hospitalisation cost of 39.9%. The average annual indirect cost was approximately four times higher for patients with hospitalisation (US$158.9) compared to that of patients without hospitalisation (US$41.8).

**Table 3:** Costs-of-illness of type 2 diabetes per person per year (in US$) by components of direct and indirect costs

Cost components	Mean	Median	25^th^p, 75th p	90th p	% of total	Total COI	% of total COI
Overall COI (*n* = 1263)							
*a. Direct cost*							
*i. Direct medical cost*					96.9		
Outpatient visit	11.8	6.3	2.2, 12.5	31.2	1.5	14,807.1	1.4
Hospitalisation^a^	216.7	496.6	0, 276.7	673.1	27.7	271,490.4	12.7
Medicine	474.5	331.0	205.3, 474.5	798.4	60.7	594,543.8	54.9
Laboratory tests	37.7	34.0	23.3, 47.2	62.1	4.8	47,311.9	16.8
Other service utilisation^#^	16.5	9.0	0, 18	54.0	2.1	20,718	1.8
*ii. Direct non-medical cost*					3.1		
Transportation	23.7	7.5	2.5, 27.5	62.5	3.0	29,748.7	2.8
Meal	0.7	0.0	0, 0	2.5	0.1	931.4	0.1
Total direct cost	781.7	453.6	276.6, 893.4	1705.0	100	979,551.3	90.5
*b. Indirect cost*							
Productivity loss of patient	67.8	26.0	0, 62.5	145.8	81.8	85,062.5	7.9
Productivity loss of accompanied person	15.1	0.0	0, 0	39.1	18.2	18,872.2	1.7
Total indirect cost	82.9	36.5	0, 82.8	187.5	100	103,934.8	9.5
Total cost	864.7	504.2	308.8, 982.7	1874.3		1,083,486.0	
Without hospital admission (*n* = 813)							
Direct cost							
*i. Direct medical cost*					93.9		
Outpatient visit	5.9	5.0	1.25, 8.75	12.5	1.6	4801.6	
Hospitalisation^a^	–	–	–	–	–	–	
Medicine	307.2	255.5	173.4, 387.8	520.1	83.5	249,827.3	
Laboratory tests	34.2	31.2	21.5, 41.8	53.9	9.3	27,828.6	
Other service utilisation^b^	10.5	9.0	0, 9	18.0	2.8	8523.0	
*ii. Direct non-medical cost*					2.8		
Transportation	9.4	3.7	2, 8.75	21.0	2.6	7662.6	
Meal	0.7	0.0	0, 0.6	2.3	0.2	524.5	
Total direct cost	367.9	320.1	227.9, 452.8	608.9	100	299,167.5	
Indirect cost							
Productivity loss of patient	34.7	24.7	0, 52.1	71.9	83.0	28,244.5	
Productivity loss of accompanied person	7.1	0.0	0, 0	20.8	17.0	5790.9	
Total indirect cost	41.8	31.3	3.9, 62.5	93.7	100	34,035.4	
Total cost	409.8	366.6	261.9, 505.7	662.7		333,202.9	
With hospital admission (*n* = 440)							
Direct cost							
*i. Direct medical cost*					96.7		
Outpatient visit	22.7	12.5	5, 37.5	51.3	1.5	10,005.5	
Hospitalisation^a^	617.0	419.4	247.3, 753.2	1303.9	39.9	271,490.4	
Medicine	783.4	469.9	346.7, 764.0	1455.4	50.7	344,716.5	
Laboratory tests	44.3	41.8	29.7, 56.9	71.6	2.9	19,483.4	
Other service utilisation^b^	10.0	9.0	9, 36	54.0	1.8	12,195.0	
*ii. Direct non-medical cost*					3.3		
Transportation	50.2	31.2	15, 62.5	112.5	3.2	22,086.1	
Meal	0.9	0.0	0, 0	2.5	0.1	406.9	
Total direct cost	1546.3	1121.1	806.3, 1845.9	2927.7	100.0	680,383.8	
Indirect cost							
Productivity loss of patient	129.1	35.1	0, 145.8	281.2	81.3	56,818.1	
Productivity loss of accompanied person	29.7	0.0	0, 0	104.1	18.7	13,081.2	
Total indirect cost	158.9	67.7	0, 182.9	345.1	100.0	69,899.4	
Total cost	1705.2	1247.4	876.9, 1996.6	3353.8		750,283.2	

^a^Hospitalisation includes hospital stay, medicine and laboratory tests during stay. ^b^Other service utilisation includes use of self-monitoring blood glucose and consumables

### Sensitivity analysis

The result of the one-way sensitivity analysis showed that use of the median income of the study participants instead of the minimum wage increased the indirect cost by 23%. However, the estimated average annual cost using the minimum wage rate of Bangladesh was US$853 (95% CI US$795.1-US$911.7), while it was US$864.7 (95% CI 806.5–922.9) using the median income of the study participants. The difference between these two estimates is insignificant as the CI overlapped each other.

The results of a two-way sensitivity analyses showed that the average annual cost increased by 2.9% (US$865 vs US$890) when insulin use was increased by 25% and that decreased by 4% (US$865 vs US$830) when insulin use was decreased by 25%. A 25% increase in prevalence of complications lead to a 5.3% (US$865 vs UD$898) increment of average annual cost, while it decreased by 3.9% (US$865 vs US$819) with a 25% reduction in complications.

### Determinants of cost-of-illness

The results of simple and multiple median regression analyses are presented in Table 4. In the simple median regression analysis, age group 61–80 years (US$221.91, *p* < 0.001) and more than 80 years (US$741.58, *p* < 0.001), treated with insulin alone (US$140.69, *p* = 0.042) as well as with a combination of OHA (US$307.38, *p* < 0.001), duration of diabetes more than 10 years (US$368.68, *p* < 0.001), poor (HbA1c ≥8%) glycaemic control (US$79.41, *p* = 0.009), presence of any complication (US$201.54 for one or two and US$287.72 for more than two, *p* < 0.001), presence of hypertension (US$254.89*p* < 0.001) alone and hypertension with a combination of dyslipidaemia (US$169.07, *p* = 0.001) were significantly associated with higher costs.

**Table 4:** Median regression analysis of total cost

Variables	Unadjusted			Adjusted		
	Coefficients	*p*-value	95% confidence interval (CI)	Coefficients	*p*-value	95% confidence interval (CI)
Gender (ref: Male)						
Female	19.03	0.492	−35.32-73.37	44.85	0.036	3.02–86.68
Age (≤40 years)						
41–60 years	82.85	0.107	−17.79-183.49	21.13	0.419	−30.13-72.39
61–80 years	221.91	< 0.001	113.60–330.24	2.86	0.930	−60.70-66.41
≥ 80 years	741.58	< 0.001	499.87–938.30	170.76	0.708	− 723.11-1064.64
Mode of treatment (ref: OHA)						
Insulin	140.69	0.042	5.28–276.09	65.40	0.260	−48.28-179.07
Insulin + OHA	307.38	< 0.001	237.90–377.65	152.87	< 0.001	107.45–198.30
Duration of diabetes (ref: ≤5 year)						
6–10	78.36	0.080	−9.42-166.15	17.59	0.403	−23.68-58.82
≥ 11	368.68	< 0.001	289.32–448.04	66.93	0.025	8.55–125.32
HbA1c (ref: ≤6.9)						
Fair (7–7.9)	45.42	0.216	−26.60-177.43	−1.20	0.949	−63.50-59.51
Poor (≥8)	79.41	0.009	20.30–138.53	22.50	0.406	−30.58-75.58
History of co-morbidity (ref: None)						
Hypertension	164.89	< 0.001	76.10–252.79	30.13	0.213	−17.25-77.51
Dyslipidaemia	−42.17	0.494	− 163.00-78.66	2.88	0.924	−65.16-61.91
Hypertension+dyslipidaemia	169.07	0.001	68.60–269.55	53.07	0.098	−9.75-115.89
Number of complication (ref: None)						
One or two	210.54	< 0.001	134.48–28,660	63.69	0.003	21.70–105.68
Three or more	847.72	< 0.001	739.82–955.63	440.93	< 0.001	274.08–607.85

Multiple regression analysis showed that the average annual cost was higher for females (US$44.85, *p* = 0.036). People treated with insulin with a combination of OHA (US$152.87, *p* < 0.001) also had higher costs compared to those treated with OHA only. Patients with a duration of diabetes of more than 10 years (US$66.93, *p* = 0.025) incurred a higher cost. Likewise, patients with the presence of any complication (US$63.69 for one or two and US$440.93 for more than two, *p* < 0.001) had higher costs compared to those without any complication.

## Discussion

Diabetes has become a major global economic burden in recent decades, but proper management of the factors related to it can be useful for reducing this burden. Diabetes is also an increasingly economic threat in Bangladesh, yet studies on an adequate estimation of COI for T2DM and its key drivers are limited; hence, the aim of this paper. This study involved a large representative sample that adequately investigated the economic burden of type 2 diabetes in Bangladesh from the patients’ perspective. The key finding of this study was that the average annual cost for T2DM patient is US$865 with the medicine cost being the highest contributor followed by the hospitalisation cost. The average annual cost for patients with hospitalisation was 4.2 times higher compared to those without hospitalisation.

The average annual cost for each person with T2DM in Bangladesh appears to be considerably higher than that reported in previous studies conducted in Bangladesh (US$314) [19] and other South Asian countries such as India (US$525) [28] and Pakistan (US$197) [29]. One possible explanation of this difference may be because the studies conducted in Bangladesh and Pakistan addressed only outpatient department cost, which underestimated the overall cost. In contrast, some high- or upper middle-income Asian countries, for example, China (US$1501.7) [30] and Singapore (US$1575.6) [18], reported higher cost for diabetes management.

This study finding showed that cost increased with age, which is supported by previous studies [14, 17, 31]. Additionally, female gender was a factor more likely to incur higher cost. A study conducted in Bangladesh by Shamima et al. showed that females had better awareness about diabetes and were more regular in receiving follow-up check-ups [7], which may be related to higher cost. A study conducted in Hawaii by Bhattacharyya et al. [14] showed a similar result, while Krop et al. [31] in Maryland and Chaikledkaew et al. [32] in Thailand showed that the cost of care was higher for males.

An important finding of this study was that 13.5% of participants had income less than the estimated average annual cost. Overall, a person with T2DM spent 9% of his/her annual household income on management, which is a notable financial burden for a family. In South Asia, health insurance is practically non-existent, and almost all expenses are met through OOP, which creates a significant burden and sometimes leads to family impoverishment. This study showed that urban residents spent more than the rural residents (mean cost for urban: US$1024.4, vs rural: US$421.6). This may be because of people residing in urban areas have better education and a higher income, and thus can better afford to receive adequate treatment and access to specialised doctors.

The present results showed that direct cost had the largest share (90.5%) of overall cost. Among all the cost components of overall direct cost, medicine cost was the major contributor (60.7%). The studies conducted by Khowaja et al. [29] in Pakistan and Shobhana et.al [28] in India reported similar features. However, compared to these studies, the present study showed a much higher proportion of medicine cost. This difference may be related to many factors. Firstly, 93% of the participants in this current study had high or medium adherence to medication, which incurs a higher medicine cost. Secondly, among them, 65.6% used either insulin alone or insulin with a combination of OHA, which is higher than that reported in another study in Bangladesh [33]. The guidelines for treatment and management of diabetes in Bangladesh follows lifestyle management as the first line care, metformin as second line care and then insulin, etc. depending on the health requirement (presence of comorbidity and complications) of the patients. Moreover, as all medical costs come from out of pocket payments, people usually visit doctor when diabetes makes obstacle to their daily living. This explained why a very low number of participants (1.8%) in this study were under lifestyle management. Thirdly, since insulin is very expensive in Bangladesh, it leads to a higher medicine cost. The result of other studies conducted in some developing countries [14, 34–37] also showed the medicine cost as a major contributor to direct cost.

Medicine was the highest source of direct cost (83.5%) for patients without hospitalisation. Furthermore, for patients with hospitalisation, medicine cost was 50.7% of the direct cost followed by a hospitalisation cost of 39.9%. However, a number of previous studies showed that the largest proportion of cost is attributable to hospitalisation followed by medicine cost. In the USA, hospitalisation cost accounted for 50% [38], while that was 55% in the Cost of Diabetes in Europe-Type II study [39].

In the present study, the cost of diabetes care substantially increased with the presence of comorbidities as well as complications related to T2DM. The cost was positively correlated with the increased number of comorbidities and complications leading to hospitalisation. This finding is supported by other studies in the developed [30, 37, 40–42] as well as in developing countries [43, 44]. In addition to complications and comorbidities, the duration of diabetes also accelerates cost; likewise, cost increased for patients who had poor glycaemic control compared to good control. Similar results were reported in previous COI studies [28, 29, 37, 45].

A Median regression analysis showed that female gender, use of insulin, longer duration of diabetes, and presence of complications are the factors related to a higher annual average cost per person. A majority of these variables also appeared as contributing factors in previous studies [14, 15, 17, 28, 42]. Thus, optimisation of the management of diabetes-related complications is an imperative need for people with T2DM in Bangladesh, which has also reflected in the results of sensitivity analyses of the current study.

The present study showed that in 2017 the annual average cost per T2DM was US$864.7, which is 52% of per capita GDP of Bangladesh [10] and 9.8 times higher than the general health care cost [9]. The burden of diabetes is influenced by many socio-economic and health care system factors, which consequently affects the cost of care. Early screening is one of the factors that may help to diagnose T2DM patients at an initial stage, thus avoiding complications. However, in developing countries, people often seek medical help when they have already developed some complications. In addition, inadequate awareness about diabetes-related complications, lack of access to medical care resulting from income disparities, lack of social supports, and heterogeneous quality of care are other societal factors that influence the diabetes-related cost of care [46]. Thus, this study finding will be useful for policy makers in planning future health care needs and allocating scarce resources. Furthermore, it will play a significant role for both patients and provider in identifying and quantifying the costs attributed to T2DM in Bangladesh.

A strength of this study was that it addressed all possible cost components of both direct (including hospitalisation) and indirect cost from the patients’ perspective, and cost was calculated based on primary data. However, other recent COI studies have not addressed all cost components [3, 38], despite the cost being calculated from the societal perspective. Another strength was that a professional mix of patients residing in urban and rural locations was recruited randomly form six hospitals that provide primary to tertiary care.

Some limitations should also be noted. Firstly, in addition to direct and indirect costs, there are also intangible costs (e.g. pain, suffering, and loss of quality of life), which was not addressed in this study. Secondly, due to a lack of information, the calculation of indirect cost was done using the traditional human capital approach rather than the frictional cost approach [47]. Finally, as it was a descriptive cost-of-illness study, it did not provide information on the efficiency of resource use; thus, higher cost does not necessarily mean better services or value for money.

## Conclusions

Diabetes is a major public health issue with a high economic burden in Bangladesh. The development and improvement of interventions toward better control of T2DM and the prevention of its complications are vital requirements for the country. Without these, in the near future, the private and public financing of diabetes treatment will be severely constrained, representing a health threat for the Bangladeshi population.

## Additional file

Additional file 1: English language versions of the questionnaire (PDF 8 kb)

## Data Availability

The data generated during and/or analysed during the current trial are available from the corresponding author on reasonable request.

## Abbreviations

BADAS: Diabetic Association of Bangladesh
BDT: Bangladeshi currency, Taka
BUHS: Bangladesh University of Health Sciences
CI: Confidence interval
COI: Cost -of-illness
GDP: Gross domestic product
IDF: International Diabetes Federation
LMICs: Low- and lower middle-income countries
OHA: Oral hypoglycaemic agent
OOP: Out-of-pocket
PPS: Probability proportional to size
REDCap: Research Electronic Data Capture
T2DM: Type 2 diabetes mellitus
WHO: World Health Organization
