# Supplementary figures and images for: Type 2 diabetes mellitus in Bangladesh: a prevalence based cost-of-illness study
Source: BMC Health Serv Res. 2019 Aug 27;19:601. doi: 10.1186/s12913-019-4440-3 (PMC6712789; doi:10.1186/s12913-019-4440-3)

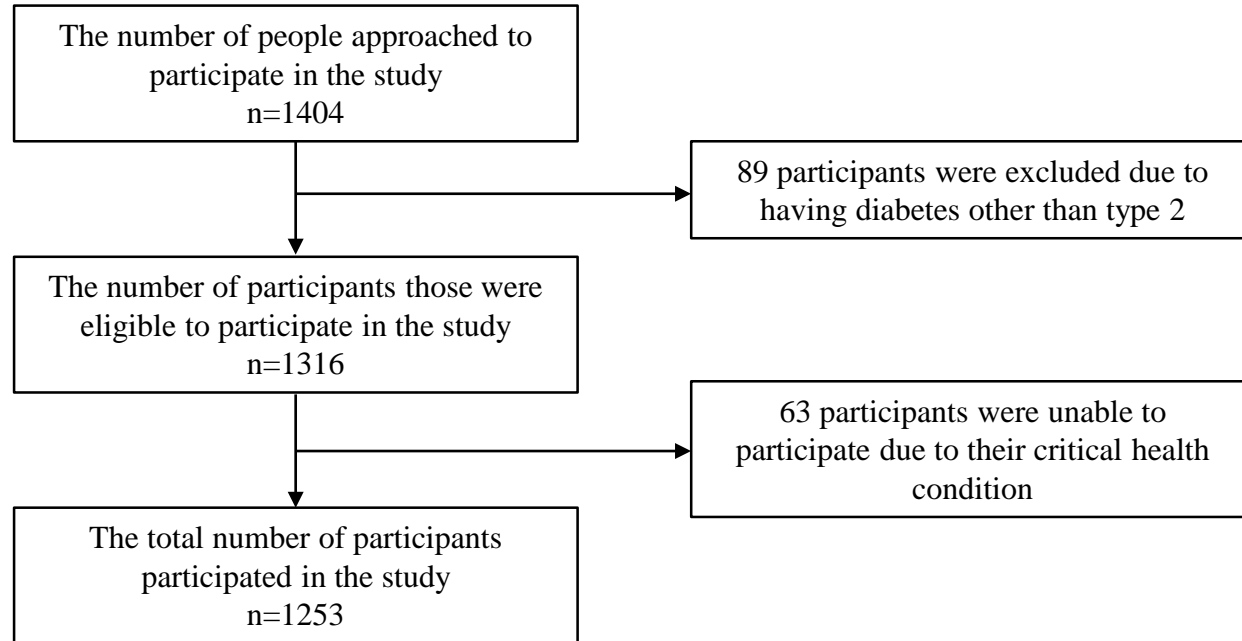

Supplement: Supplementary file 1 — English language versions of the questionnaire (PDF 8 kb) [file 12913_2019_4440_MOESM1_ESM.pdf]
